# Supplementary material for: Drosophila AHR limits tumor growth and stem cell proliferation in the intestine
Source: Wellcome Open Res. 2025 Apr 25;10:38. Originally published 2025 Feb 3. [Version 3] doi: 10.12688/wellcomeopenres.23515.3 (PMC11982807; doi:10.12688/wellcomeopenres.23515.3)
Supplement: Peer review history

# Reviewer report: Reviewer response for version 2

**DOI:** 10.21956/wellcomeopenres.26499.r121263

**Published:** 2025-04-09

**Authors:** Palmira Barreira-Silva

**Affiliations:** 1 Universidade do Porto Instituto de Biologia Molecular e Celular (Ringgold ID: 89238), Porto, Porto District, Portugal

In this work, Tsai *et al* focus on studying the potential role(s) of Spineless, a *Drosophila* ortholog of the Aryl Hydrocarbon Receptor (AHR), in adult intestine homeostasis and regeneration. The authors demonstrate a new and conserved role for Spineless in controlling intestinal stem cell (ISC) proliferation and differentiation in the adult *Drosophila* midgut. Using disease models to investigate the functional consequences of Spineless expression, the authors showed that the overexpression of Spineless decreased the proliferation of ISC, reducing the survival of *Pseudomonas entomophila*-infected flies. Additionally, the authors showed that in two different tumour models, the overexpression of Spineless limited tumour growth, potentially by restraining ISC proliferation and promoting cell differentiation, increasing survival of the tumour-affected flies.

 In general, this is a well-designed and clear study unravelling evolutionary conserved functions of the AHR/Spineless in intestine epithelial homeostasis, that go beyond the role of this AHR ortholog in development. This emphasises the potential of using the *Drosophila* model to study the roles of AHR/Spineless in disease and homeostasis conditions.

**Please address some review requests and comments that I believe will benefit the manuscript:**

1. In figure legends, when the authors state that “data is from one experiment”, does this mean that the experiment depicted is representative of other replicates or was the experiment performed only once? The case of experiments being performed only once might weaken the conclusions drawn from them.
2. The reference to Figure 5 panels I to N in the text is not correct, please rectify this on pages 13 and 17.

Is the work clearly and accurately presented and does it cite the current literature?

Yes

If applicable, is the statistical analysis and its interpretation appropriate?

Yes

Are all the source data underlying the results available to ensure full reproducibility?

Yes

Is the study design appropriate and is the work technically sound?

Partly

Are the conclusions drawn adequately supported by the results?

Partly

Are sufficient details of methods and analysis provided to allow replication by others?

Yes

Reviewer Expertise:

Immune responses to infectious diseases

I confirm that I have read this submission and believe that I have an appropriate level of expertise to confirm that it is of an acceptable scientific standard.

# Reviewer report: Reviewer response for version 2

**DOI:** 10.21956/wellcomeopenres.26499.r121265

**Published:** 2025-04-09

**Authors:** Ben Ohlstein

**Affiliations:** 1 Children's Medical Center Research Institute, Texas, USA

I’ve read the other reviewer’s comments and agree with them.

**I only have a few comments that should be addressed:**

1. The authors state that “In the midgut, we could not detect a clear GFP signal in naïve or P. entomophila infected ssGFP flies (Figure 1I, J).” I was curious to know why GFP was not detected and how that might affect the results.
2. “we generated ssGFP, esgts>anti-GFP flies.” I may have missed this. But what is the advantage of using this approach as opposed to RNAi. I’m guessing it may be because the approach could result in a quicker inactivation of spineless.
3. What does knockdown and overexpression of spineless do to PH3+ in the absence of PE?
4. The manuscript depends on Gal4 inactivation and expression. Infrequent clones of spineless mutants (lof, UAS-RNAi) and clones overexpressing spinless would make it a lot easier to draw conclusions on their effects on proliferation and lineage differentiation.

Is the work clearly and accurately presented and does it cite the current literature?

Yes

If applicable, is the statistical analysis and its interpretation appropriate?

Yes

Are all the source data underlying the results available to ensure full reproducibility?

Yes

Is the study design appropriate and is the work technically sound?

Yes

Are the conclusions drawn adequately supported by the results?

Partly

Are sufficient details of methods and analysis provided to allow replication by others?

Yes

Reviewer Expertise:

Developmental Biology, Stem Cell Biology

I confirm that I have read this submission and believe that I have an appropriate level of expertise to confirm that it is of an acceptable scientific standard, however I have significant reservations, as outlined above.

# Reviewer report: Reviewer response for version 1

**DOI:** 10.21956/wellcomeopenres.25936.r118155

**Published:** 2025-02-25

**Authors:** Jin Jiang, Mengjie Li

**Affiliations:** 1 UT Southwestern Medical Center, Dallas, Texas, USA; 2 Department of Molecular Biology, UTSW, Dallas, USA

Mammalian aryl hydrocarbon receptor (AHR) regulates intestine homeostasis and regeneration but whether the Drosophila homolog of AHR, Spineless (SS), plays a conserved role in midgut homeostasis and regeneration has remained unexplored. In this study, Tsai et al investigated the role of SS in the regulation of intestinal stem cell (ISC) proliferation and differentiation in adult *Drosophila *midgut. The authors found that bacterial (PE) infect dramatically increased the expression of SS, which is consistent with a previous study showing that PE increased SS expression in ISC/EB population. Furthermore, they found that overexpression of SS limited ISC proliferation in PE infected guts and reduced the survival of the infected flies whereas inactivation of SS increased ISC proliferation in PE infected guts. The growth suppression function of SS was further examined in N ^RNAi ^and Yki ^act^ midguts, in which overexpression of SS dramatically suppressed tumor growth and increased the survival of tumor-bearing flies. To determine how SS inhibited tumor growth, the authors carried out RNA seq experiments and found that overexpression of SS suppressed DNA replication genes and Stat92E while increasing the expression of genes involved in EE differentiation genes in esg>GFP ^low^ (putative ISC cells). In addition, the authors found that overexpression of SS reversed the effect of Yki ^act^ on the expression of genes involved in cell proliferation and metabolism. Taken together, these results suggest that Spineless can act as a tumor suppressor in the adult *Drosophila* midgut by limiting ISC proliferation and promoting cell differentiation. This study reveals a potential tumor suppressor function of SS in Drosophila adult intestine although the conclusion is derived mainly from overexpression study and the precise mechanisms remain to be determined. The study also provides extensive sequencing datasets, which could improve the understanding of the differences in gene expression between ISC and EB. That said, the manuscript can be improved by addressing the concerns indicated below.

**Major concerns**

 1. A major weakness of this study is that the authors drew their conclusions almost entirely from overexpression study, leaving the physiological role of SS in Drosophila midgut explored. Indeed, according to their own measurement, *esgts>ssHA* achieved  >10,000-fold increase in SS expression over the endogenous level and ~50-fold increase over the level induced by PE. At such a high level, SS might regulate cellular events that it may not do normally under physiological or even pathological condition, which is an important caveat that needs to be acknowledged.

 2. It is interesting that SS is induced by bacterial infection and the authors suggest that SS may play a role in driving intestine back to homeostasis after repair, which is in line with the role that AHR is thought to play in mammalian intestine. The authors should test this hypothesis by examining how inactivation of SS affects the return of ISC to homeostasis post infection. If necessary, the authors could test this in other injury models such as bleomycin or DSS treatment that may provide a better system to study recovery after acute injury.

 3. Another way of revealing possible loss-of-function phenotype is to determine whether inactivation of SS could enhance ISC proliferation caused by esg>Yki ^act  ^or esg>Yki (it might be easier to modify the ISC overproliferation phenotype caused by esg>Yki because it is weaker).

**Other concerns:**

 4. Given the proposed role of SS in inhibiting ISC proliferation, it is puzzling that SS overexpression increased *esg>GFP* positive cells in uninfected guts (Figure 2A top panels; Fig. 2H, I). Does SS overexpression block EB to EC differentiation so that progenitor cells accumulate under this condition? On the other hand, immunostaining clearly showed a dramatic reduction of esg>GFP+ cells caused by SS overexpression in PE infected guts (Fig. 2A bottom panels); however, this not reflected by the results of FACS sorting experiments (Fig. 2H and I).

 5. The authors used FACS sorting to isolate ISCs and EBs based on differential esg>GFP expression (esg>GFP ^low^ for ISC and esg>GFP ^high^ for EB). Can they immunostain esg>GFP expressing guts with ISC and EB markers to confirm that EB does express higher esg>GFP than ISC. It would be also informative to provide the entire list of differentially expressed genes identified by their RNA seq in esg>GFP ^low^ and esg>GFP ^high ^cells as a supplementary table and compare the list with published lists of ISC- and EB-enriched genes identified by other groups in order to boost the confidence that esg>GFP ^low^ and esg>GFP ^high ^do faithfully mark the ISC and EB, respectively.

 6.  Figure 3. It is striking that SS overexpression almost completely suppressed ISC-like tumor growth in N ^RNAi ^guts. However, it is unclear whether this is due to inhibition of ISC proliferation or ISC differentiation into EE (or EB). To distinguish these possibilities, the authors should examine pHH3, ISC, EB and EE markers in these guts by immunostaining 

 7. Figure 2A, it seems that esg-GFP+ cells in naïve flies with SS overexpression have larger size compared with control guts. However, in Figure 2J, K, Q and S, SS overexpression resulted in a reduction in cell size.

 8. Figure 2, the duration of P.E infection treatment needs to be more clearly indicated.

 9. Why EB>GFP survived less well than ISC>GFP after PE infection (Figure. 2P)

 10. The authors carried out many FACs sorting experiments (Fig. 2B-T; Fig. 4D-M). It would be informative to indicate how much material (number of guts) they used for individual experiments in the figure legends.

 11. The authors used “knockdown” normally used to refer to RNAi experiments to describe their approach to inactivate SS by sequestering it in the cytoplasm. To avoid confusion, they should change “knockdown” to another word such as “inactivation.”

 12 *.* *“* *The transcriptional coactivator yorkie can regulate ISC proliferation during midgut epithelial regeneration* *^39^* *^,^* *^47^* *”*

*--*The authors should also cite two other studies (shown below) that came out at the same time as refs 39 and 47.

Is the work clearly and accurately presented and does it cite the current literature?

Yes

If applicable, is the statistical analysis and its interpretation appropriate?

Yes

Are all the source data underlying the results available to ensure full reproducibility?

Yes

Is the study design appropriate and is the work technically sound?

Partly

Are the conclusions drawn adequately supported by the results?

Partly

Are sufficient details of methods and analysis provided to allow replication by others?

Yes

Reviewer Expertise:

Developmental genetics

We confirm that we have read this submission and believe that we have an appropriate level of expertise to confirm that it is of an acceptable scientific standard, however we have significant reservations, as outlined above.

## References

[1] : The Hippo tumor suppressor pathway regulates intestinal stem cell regeneration. *Development* .2010;137(24) : 10.1242/dev.060483 4135-45 10.1242/dev.060483 21098564 PMC2990205

[2] : Hippo signaling regulates Drosophila intestine stem cell proliferation through multiple pathways. *Proc Natl Acad Sci U S A* .2010;107(49) : 10.1073/pnas.1012759107 21064-9 10.1073/pnas.1012759107 21078993 PMC3000252

# Response

**Published:** 2025-03-18

**Authors:** Nicola Laura Diny

**Affiliations:** University of Bonn, Bonn, Germany

**Reviewer Report**   Jin Jiang, UT Southwestern Medical Center, Dallas, Texas, USA Mengjie Li, Department of Molecular Biology, UTSW, Dallas, USA   25 Feb 2025 Version 1  

**Comment:** Mammalian aryl hydrocarbon receptor (AHR) regulates intestine homeostasis and regeneration but whether the Drosophila homolog of AHR, Spineless (SS), plays a conserved role in midgut homeostasis and regeneration has remained unexplored. In this study, Tsai et al investigated the role of SS in the regulation of intestinal stem cell (ISC) proliferation and differentiation in adult *Drosophila *midgut. The authors found that bacterial (PE) infect dramatically increased the expression of SS, which is consistent with a previous study showing that PE increased SS expression in ISC/EB population. Furthermore, they found that overexpression of SS limited ISC proliferation in PE infected guts and reduced the survival of the infected flies whereas inactivation of SS increased ISC proliferation in PE infected guts. The growth suppression function of SS was further examined in N ^RNAi ^and Yki ^act^ midguts, in which overexpression of SS dramatically suppressed tumor growth and increased the survival of tumor-bearing flies. To determine how SS inhibited tumor growth, the authors carried out RNA seq experiments and found that overexpression of SS suppressed DNA replication genes and Stat92E while increasing the expression of genes involved in EE differentiation genes in esg>GFP ^low^ (putative ISC cells). In addition, the authors found that overexpression of SS reversed the effect of Yki ^act^ on the expression of genes involved in cell proliferation and metabolism. Taken together, these results suggest that Spineless can act as a tumor suppressor in the adult *Drosophila* midgut by limiting ISC proliferation and promoting cell differentiation. This study reveals a potential tumor suppressor function of SS in Drosophila adult intestine although the conclusion is derived mainly from overexpression study and the precise mechanisms remain to be determined. The study also provides extensive sequencing datasets, which could improve the understanding of the differences in gene expression between ISC and EB. That said, the manuscript can be improved by addressing the concerns indicated below.  

**Author response: **We thank the reviewers for their thorough assessment of our manuscript and for their comments and suggestions which have helped to clarify aspects of our manuscript. We have updated our manuscript upon their suggestions as we explain below in response to each comment.

**Major concerns:**

**Comment 1: ** A major weakness of this study is that the authors drew their conclusions almost entirely from overexpression study, leaving the physiological role of SS in Drosophila midgut explored. Indeed, according to their own measurement, *esgts>ssHA* achieved  >10,000-fold increase in SS expression over the endogenous level and ~50-fold increase over the level induced by PE. At such a high level, SS might regulate cellular events that it may not do normally under physiological or even pathological condition, which is an important caveat that needs to be acknowledged.

**Author response:** We agree with the reviewers that this is an important limitation of our study and have highlighted this further in the discussion section: “A limitation of this study is that the findings on the tumour suppressor functions of Spineless are largely based on overexpression studies. We did observe minor effects of Spineless inactivation, which lead to accelerated death in the Notch RNAi tumour model. The effects of Spineless overexpression, however, were much more pronounced and further explored in the yki act tumour model and the RNA sequencing data. It is possible that overexpression of Spineless at such high levels results in the interaction with novel cellular targets and therefore novel phenotypes.”

**Comment 2:** It is interesting that SS is induced by bacterial infection and the authors suggest that SS may play a role in driving intestine back to homeostasis after repair, which is in line with the role that AHR is thought to play in mammalian intestine. The authors should test this hypothesis by examining how inactivation of SS affects the return of ISC to homeostasis post infection. If necessary, the authors could test this in other injury models such as bleomycin or DSS treatment that may provide a better system to study recovery after acute injury.  

**Author response: **We thank the reviewers for this interesting suggestion to further compare the AHR and Spineless functions across species. In preliminary experiments we have tested several stimuli for their ability to induce spineless expression and could not detect any induction of spineless after treatment with 3-5% DSS in sucrose for 4h, 24h or 48h as compared to controls. Therefore, DSS injury might not be a suitable model system to study the effects of Spineless on epithelial injury and repair. We have not conducted experiments to determine the ISC proliferation in flies with inactivated Spineless at later timepoints after P. e. infection to determine if the phenotype of increased proliferation is retained beyond 24h. We have acknowledged in the discussion that this should be tested with further experiments to compare this function across species: “In mammals, AHR is needed to end the regenerative response of the intestinal epithelium after injury to allow the epithelial barrier to return to its mature state ^5^ . Whether Spineless fulfils similar functions by limiting ISC proliferation and promoting differentiation into mature epithelial cells after bacterial infection or in tumour models remains to be tested in flies with Spineless inactivation or knockdown.”

**Comment 3:** Another way of revealing possible loss-of-function phenotype is to determine whether inactivation of SS could enhance ISC proliferation caused by esg>Yki ^act  ^or esg>Yki (it might be easier to modify the ISC overproliferation phenotype caused by esg>Yki because it is weaker).

**Author response:** The esg>Yki ^act^ modification indeed leads to very rapid overproliferation of progenitor cells and a median survival of the flies of 7 days (Figure 4B). Using the esgts>GFP.NLS construct, overproliferation of progenitor cells was already present at day 2 as we could observe by both microscopy and flow cytometry (Figure 4C, G, H). We agree with the reviewer that it could be difficult to observe a further induction of ISC proliferation by Spineless inactivation in this context. We did not assess the effect of Spineless inactivation in either the esg>Ykiact  or esg>Yki models. We have acknowledged this limitation of our manuscript as described above.

**Other concerns:**

**Comment 4:** Given the proposed role of SS in inhibiting ISC proliferation, it is puzzling that SS overexpression increased *esg>GFP* positive cells in uninfected guts (Figure 2A top panels; Fig. 2H, I). Does SS overexpression block EB to EC differentiation so that progenitor cells accumulate under this condition? On the other hand, immunostaining clearly showed a dramatic reduction of esg>GFP+ cells caused by SS overexpression in PE infected guts (Fig. 2A bottom panels); however, this not reflected by the results of FACS sorting experiments (Fig. 2H and I).  

**Author response: **We thank the reviewers for their careful analysis of this figure. Indeed, both the microscopy and flow cytometry data in Figure 2A, H, I suggest an increased number of esg>GFP positive cells in midguts from spineless overexpressing flies. In combination with our data on stem cell proliferation (Figure 1P), which shows no difference in proliferation in uninfected flies, this would suggest a potential delay or block in differentiation of EB to EC. In the results, we discuss also the increase in esg>GFP fluorescent intensity caused by spineless overexpression within the GFP-high cells (Figure 2N) and the increased escargot expression as shown by qPCR of sorted cells (Figure 2O). This suggests that spineless affects escargot expression within enteroblasts, which was shown to maintain stemness. This could be a potential mechanism of how spineless blocks differentiation of EB to EC.  Following P.e. infection, we see a substantial increase in the size of esg>GFP positive cells, in particular in the esg>GFP high cells (Figure 2J, K), which is also visible on the fluorescent images in Figure 2A. Of note, cell size is reduced in flies with spineless overexpression. This discrepancy in cell size could account for some of the differences in the GFP fluorescence observed by microscopy as larger cells cover a bigger area on the fluorescent images and it is not straight forward to discriminate cell size versus number on images. Furthermore, we cannot exclude that larger cells and in particular fused cells could be prone to increased cell death during the processing of the midguts for flow cytometry, which involves enzymatic and mechanic dissociation. We note a large variability in the flow cytometry data on cell numbers (in Figures 2H, I) as opposed to measures on cell size and fluorescent intensity (Figure 2J-N). It is possible that increased death during processing resulted in a larger number of cells being lost from the control flies compared to the spineless overexpressing flies. We have now added this potential caveat to the results section: “We note a large variability in the normalized cell numbers, which may result from differential susceptibility to cell death of large or fused cells during midgut processing.”

**Comment 5:** The authors used FACS sorting to isolate ISCs and EBs based on differential esg>GFP expression (esg>GFP ^low^ for ISC and esg>GFP ^high^ for EB). Can they immunostain esg>GFP expressing guts with ISC and EB markers to confirm that EB does express higher esg>GFP than ISC. It would be also informative to provide the entire list of differentially expressed genes identified by their RNA seq in esg>GFP ^low^ and esg>GFP ^high ^cells as a supplementary table and compare the list with published lists of ISC- and EB-enriched genes identified by other groups in order to boost the confidence that esg>GFP ^low^ and esg>GFP ^high ^do faithfully mark the ISC and EB, respectively.  

**Author response: **We thank the reviewers for their suggestions. We provide several indications that GFPlow cells may correspond to ISC and GFPhigh cells may correspond to EB. GFPhigh cells are larger in cell size than GFPlow cells (Figure 2F), as would be expected for EB. As is shown in Figure 2G, the stem cell marker delta shows higher expression in GFPlow than GFPhigh cells. On the level of pathway enrichment, we can find distinct functional difference between GFPhigh and GFPlow cells as is shown in Figure 5G. GFP ^low^ cells were enriched for genes relating to DNA replication, which is indicative of cell division,  Ion channel activity, and GPCR activity ( Figure 5G). GFP ^high^ cells were enriched for genes relating to ribosomes, defense response against bacteria, and metabolic pathways including fatty acid beta oxidation and aerobic respiration. They also showed increased expression of genes relating to WNT and HIPPO signaling as well as cilium morphogenesis, suggesting differentiation towards mature epithelial cells as could be expected from enteroblasts. Wellcome Open Research does not allow for supplemental Tables or Figures, therefore we cannot provide a supplemental Table with all the differentially expressed genes. All sequencing data are publicly available under GSE229388 (https://www.ncbi.nlm.nih.gov/geo/query/acc.cgi?acc=GSE229388).  In addition, we have added a table of the top 50 up- and down-regulated genes to the manuscript (see new Table 4). We hope this satisfies the reviewers’ request for presentation of additional data. We have also compared our datasets to published gene signatures of ISC and EB from the single cell RNA-sequencing dataset by Hung et.al. (www.pnas.org/cgi/doi/10.1073/pnas.1916820117). We used the marker genes for ISC and EB they report in supplemental table 6 and found that EB markers were significantly enriched in our comparison of GFPhigh versus GFPlow cells. We could not find significant enrichment of the ISC marker genes. We have updated Figure 5H and the corresponding results section with this data as well as the methods section with the details.  

**Comment 6:**  Figure 3. It is striking that SS overexpression almost completely suppressed ISC-like tumor growth in N ^RNAi ^guts. However, it is unclear whether this is due to inhibition of ISC proliferation or ISC differentiation into EE (or EB). To distinguish these possibilities, the authors should examine pHH3, ISC, EB and EE markers in these guts by immunostaining.   

**Author response:** We agree with the reviewers that a confirmation of our findings on the level of gene expression by functional experiments would be desirable. Unfortunately, this was beyond the scope of our study, and we acknowledge this shortcoming in the discussion. “Our results support two potential tumour-suppressing mechanisms for Spineless: the ability to restrain stem cell proliferation as observed following P. entomophila infection and to promote epithelial differentiation as highlighted by the transcriptomic data. Evidence for Spineless suppressing proliferation was also visible on the mRNA level, where it reversed yki act -induced pathways relating to DNA replication and cell division. Spineless largely reversed the effects of yki act on gene expression and thereby restored normal cell function and metabolism in midgut progenitors. Further experiments are needed to confirm these changes in gene expression on the functional level.”

**Comment 7: **Figure 2A, it seems that esg-GFP+ cells in naïve flies with SS overexpression have larger size compared with control guts. However, in Figure 2J, K, Q and S, SS overexpression resulted in a reduction in cell size.  

**Author response: **Our flow cytometric data in Figure 2J, K indeed show that cell size is smaller in esg>GFP positive cells of flies with spineless overexpression. Our data in Figure 2L, N also show that spineless overexpression results in a brighter GFP signal in GFPhigh cells. In the fluorescent images in Figure 2A this increase in brightness is obvious as well, in particular in the left images of the surface of the midgut. In the right images depicting transverse sections of the midgut, cell size can be more accurately judged and there is no increased size in spineless overexpressing flies. Rather, the brightness of the cells gives an impression of increased cell size in the left images.

**Comment 8:**  Figure 2, the duration of P.E infection treatment needs to be more clearly indicated.  

**Author response:** Figure 2B-G are data from naïve flies, we have added this information to the figure legend. For the remaining parts of Figure 2, the timepoint is indicated either on the graph directly or in the figure legend. In Figure 2A, L, the timepoint (24h) is indicated in the Figure itself. For Figure 2 H-N and Q-T we state in the Figure legend that midguts were analysed at 24h post infection.  For the survival curves in Figure 2P, the timepoints are indicated on the x-axis. 

**Comment 9:** Why EB>GFP survived less well than ISC>GFP after PE infection (Figure. 2P)  

**Author response: **In Figure 2P, we show the survival of flies with overexpression of spineless in either ISC or EB and the corresponding controls. In both cases, overexpression of spineless reduced the lifespan of P.e. infected flies as compared to their respective controls. We are unsure as to why the control flies showed differences in survival. It is possible that the different genetic constructs  ( esg-Gal4, Su(H)-Gal80, tub-Gal80 ^ts^ for ISC or  Su(H)-Gal4, tub-Gal80 ^ts^ for EB) could affect survival following P.e. infection.

**Comment 10:** The authors carried out many FACs sorting experiments (Fig. 2B-T; Fig. 4D-M). It would be informative to indicate how much material (number of guts) they used for individual experiments in the figure legends.  

**Author response:** We thank the reviewers for noticing the missing information. We have now stated in the methods section that n=2 midguts were pooled per sample for flow cytometric analysis, unless otherwise noted. We have also added this information to the figure legends in Figures 2 and 4. 

**Comment 11:** The authors used “knockdown” normally used to refer to RNAi experiments to describe their approach to inactivate SS by sequestering it in the cytoplasm. To avoid confusion, they should change “knockdown” to another word such as “inactivation.”  

**Author response:** We thank the reviewers for this suggestion to improve the clarity of the manuscript. We have added an explanation to the text: “Flies expressing a membrane-anchored anti-GFP nanobody ( UAS-anti-GFP) were created to prevent ssGFP from translocating to the nucleus and thereby render it inactive ( Figure 1K). To achieve cell type-specific Spineless inactivation, we utilized the GAL4/UAS system.” We changed “knockdown” to “inactivation” throughout the manuscript.

**Comment 12:** *“The transcriptional coactivator yorkie can regulate ISC proliferation during midgut epithelial regeneration ^39,47^”*

*--*The authors should also cite two other studies (shown below) that came out at the same time as refs 39 and 47.  

**Author response: **We thank the reviewers for pointing out these relevant references and have added them to the manuscript.

# Reviewer report: Reviewer response for version 1

**DOI:** 10.21956/wellcomeopenres.25936.r118147

**Published:** 2025-02-11

**Authors:** Julia B. Cordero

**Affiliations:** 1 University of Glasgow, Glasgow, UK

This study reports a functional role of Drosophila Spineless (SS) in the adult intestine, and reveals conservation with the known function of the mammalian orthologue of SS,  AHR. SS/AHR acts as a negative regulator of ISC proliferation upon infection and tumor suppressor in Yki/Yap and Notch tumors 

 Overall the study is rigorous and well designed. However, I would  request that the survival of animals in non infected conditions need to be presented for the different genotypes analysed in the paper. This is necessary to properly support the infection specific effect on survival by SS.

Is the work clearly and accurately presented and does it cite the current literature?

Yes

If applicable, is the statistical analysis and its interpretation appropriate?

Yes

Are all the source data underlying the results available to ensure full reproducibility?

Yes

Is the study design appropriate and is the work technically sound?

Partly

Are the conclusions drawn adequately supported by the results?

Partly

Are sufficient details of methods and analysis provided to allow replication by others?

Yes

Reviewer Expertise:

Intestinal stem cells; Drosophila; interurban signaling; intestinal regeneration; tumourigenesis

I confirm that I have read this submission and believe that I have an appropriate level of expertise to confirm that it is of an acceptable scientific standard.

# Response

**Published:** 2025-03-18

**Authors:** Nicola Laura Diny

**Affiliations:** University of Bonn, Bonn, Germany

**Reviewer Report** Julia B. Cordero, University of Glasgow, Glasgow, UK 11 Feb 2025 Version 1

**Comment:** This study reports a functional role of Drosophila Spineless (SS) in the adult intestine, and reveals conservation with the known function of the mammalian orthologue of SS,  AHR. SS/AHR acts as a negative regulator of ISC proliferation upon infection and tumor suppressor in Yki/Yap and Notch tumors 

 Overall the study is rigorous and well designed. However, I would request that the survival of animals in non infected conditions need to be presented for the different genotypes analysed in the paper. This is necessary to properly support the infection specific effect on survival by SS.  

**Author response: **We thank the reviewer for their assessment of our manuscript and the helpful comment. In response we have added the following results: We initially tested the P. e. infection model on w1118 flies. We found that while the median survival of infected flies (n=155) was about 2 days, we observed no deaths in flies on sucrose control (n=39) until day 6, the endpoint of the experiment. We have added this information to the methods section. Since we observed no death in the sucrose control flies, we did not include uninfected controls for each genotype in our experiments. In addition, the incubation at 29°C per se does not induce death within 6 days in any of the strains used, as can be seen in the non-tumour control groups in Figure 3B, E and Figure 4B. Therefore, we assume that uninfected flies of all genotypes used generally survive the incubation at 29°C for 6 days.
